# Analysis of the Diffusion in a Multilayer Structure under a Constant Heating Rate: The Calculation of Activation Energy from the In Situ Neutron Reflectometry Measurement

**DOI:** 10.1021/acsomega.3c04029

**Published:** 2023-07-21

**Authors:** Fuqian Yang, Harald Schmidt, Erwin Hüger

**Affiliations:** † Materials Program, Department of Chemical and Materials Engineering, University of Kentucky, Lexington, Kentucky 40506, United States; ‡ Clausthal Centre of Material Technology, Clausthal University of Technology, Clausthal-Zellerfeld DE-38678, Germany; § Solid State Kinetics Group, Institute of Metallurgy,, Clausthal University of Technology, Clausthal-Zellerfeld DE-38678, Germany

## Abstract

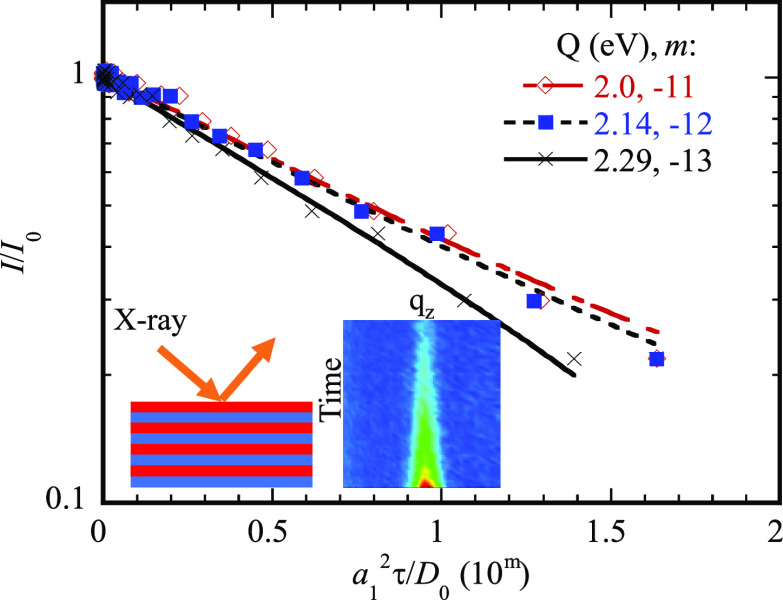

Understanding mass transport in micro- and nanostructures is of paramount importance in improving the performance and reliability of the micro- and nanostructures. In this work, we solve the diffusion problem in a multilayer structure with periodic conditions under a constant heating rate via a Fourier series. Analytical relation is established between the coefficients of eigenfunctions and the intensity of X-ray or neutron Bragg peak. The logarithm of temporal variation of the intensity of X-ray or neutron Bragg peak is a linear function of the nominal diffusion time, with the nominal diffusion time being dependent on the heating rate. This linear relation is validated by experimental data. The Taylor series expansion of the linear relation to the first order of the diffusion time yields an approximately linear relation between the logarithm of temporal variation of the intensity of X-ray or neutron peak and the diffusion time for small diffusion times, which can be likely used to calculate the activation energy for the diffusion in a multilayer structure. The validation of such an approach is subjected to the fact that the characteristic time for heat conduction is much less than the characteristic time for the ramp heating as well as the characteristic time for diffusion.

## Introduction

Understanding atomic diffusion in solids^1^ is of practical importance in controlling the synthesis of micro- and nanostructures and the performance and stability of nanoelectronic devices and systems. For example, lithium-ion diffusion is essential for the operation and integrity of a lithium-ion battery.^2−4^ In general, atomic diffusion is a thermally activated process, and the Arrhenius relation can be used to describe the temperature dependence of the diffusion coefficient of atomic diffusion as^1^
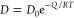
1where *D* is the diffusion coefficient, *D*_0_ is a prefactor, *Q* is the activation energy, *R* is the gas constant, and *T* is the absolute temperature.

There are various techniques available to measure the diffusion coefficient of atomic diffusion in solids. The most common technique is the tracer method,^5−10^ which tracks the spatial distribution of isotopic elements at different times under the isothermal condition with different techniques, including secondary ion mass spectrometry (SIMS).^11−20^ Using nuclear magnetic resonance (NMR)^21−24^ or quasi-elastic neutron scattering (QENS)^25−27^ makes it possible to analyze atomic diffusion in crystalline materials and in battery research. Diffusion coefficients of atomic diffusion have also been measured by low-angle X-ray diffraction (XRD) (X-ray reflectometry, XRR) in solids,^28,29^ XRD,^30,31^ energy-dispersive X-ray spectroscopy,^28,32,33^ and low-angle neutron diffraction (neutron reflectometry, NR).^34−54^ Most studies reported in the literature have been based on the isothermal condition, which require multiple measurements at different temperatures in order to determine the activation energy for atomic diffusion in solids. For example, Mizoguchi and Murata^29^ conducted low-angle XRD (XRR) analysis of compositionally modulated amorphous Co–Zr films isothermally annealed consecutively at different temperatures in a range of 120–180 °C and calculated the activation energy for the interdiffusion in the modulated amorphous Co–Zr films from the variation of the intensity of the X-ray peak.

Diffusion coefficients and activation energies for self-diffusion have been obtained with NR^37−54^ in a variety of materials. In nonoxide high-temperature superhard ceramic coatings, nitrogen self-diffusion was measured in amorphous Si_3_N_4_^35,37^ and in silicon carbo-nitrides.^38,39^ For crystalline metal oxides, lithium self-diffusion was examined in LiNbO_3_.^40,41^ Self-diffusion was measured in metals such as in nanocrystalline copper,^42^ iron,^43,44^ and Fe-based compounds.^37^ In semiconductors, self-diffusion was measured in crystalline silicon^45,46^ and germanium^47^ and in amorphous silicon^48^ and germanium.^49^ Extrinsic diffusion (so-called “Fremddiffusion”) was also suitably measured with NR^50−54^ for lithium permeation through amorphous silicon,^50−52^ amorphous lithium silicide,^53^ and nanocrystalline chromium^54^ thin layers. The diffusivities determined by NR were found to be in agreement with the corresponding ones determined from other established techniques such as SIMS.^40,41,46,47,52^ The diffusivities and the activation energies for atomic diffusion were generally measured under isothermal-diffusion annealing conditions. The multi-isothermal annealing for the determination of the activation energy of atomic motion is time-consuming and can last more than several hours.^49^

To substantially reduce the time needed in the measurement of the temperature dependence of atomic diffusion, Hüger et al.^55^ introduced the concept of increasing temperature at a constant rate in-situ NR measurements for the determination of the temperature dependence of atomic diffusion in solids. They have demonstrated the feasibility of this method in the study of atomic diffusion in amorphous ^73^Ge/^nat^Ge multilayers.^55^ In the heart of this concept is the curve fitting of the intensity of Bragg peaks with a fitting parameter of the activation energy, *Q*, as^55^
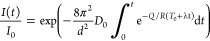
2where *I*(*t*) and *I*_0_ are the intensities of the Bragg peak at time *t* and initial time (*t* = 0), respectively, *d* is the bilayer thickness, *T*_0_ is the onset temperature, and λ is the heating rate (the increasing rate of temperature). It needs to be pointed out that eq 2 is a semi-empirical relation and the integration presented in eq 2 complexes the fitting process. Developing a simple polynomial formulation would make it readily to determine the activation energy from the temporal evolution of the intensity of the Bragg peak. Also, Hüger et al.^55^ and Schmidt et al.^37^ did not provide detailed information on the derivation of eq 2 theoretically as well as any numerical calculation of the concentration evolution during the diffusion annealing of a multilayer structure.

**Figure 1:**
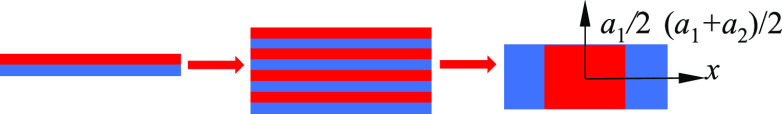
Multilayer structure consisting of a stack of a bilayer structure.

Considering the important role of atomic diffusion in the synthesis of nanostructured materials and in the structural integrity of multilayer structures, we analyze one-dimensional diffusion problem in a multilayer structure and introduce the concept of nominal characteristic time. The multilayer structure consists of a stack of a bilayer structure, as shown in Figure 1, and experiences uniform diffusion heating at a constant heating rate. The effect of the heating rate on the spatiotemporal evolution of the concentration of the diffusive species is illustrated. A polynomial equation is proposed for the determination of the activation energy of the diffusive species for the in-situ measurement of atomic diffusion by X-ray and neutron reflectometry. The lower and upper bounds of the heating rate are discussed.

## Results

Figure 1 shows a multilayer structure consisting of a stack of a bilayer structure. The thickness of each individual layer of the bilayer structure is *a*_1_ for the first layer and *a*_2_ for the second layer, as shown in the figure. Practically, both the layers in the bilayer structure likely contain diffusive species of different amounts (concentrations) before the start of diffusion, with one layer having a higher concentration of diffusive species and the other having a lower concentration of diffusive species. Without loss of generality, we assume that, at the onset of diffusion, there is uniform concentration, *c*_0_, of the diffusive species present in the first layer, and no diffusive species is present in the second layer. Note that the analysis method presented here can be readily extended to the case with uniform concentration, *c*_0_, of the diffusive species presented in the second layer and no diffusive species in the second layer. The multilayer structure experiences uniform diffusion heating under a constant heating rate of λ, and the diffusivity of the diffusive species according to eq 1 can be expressed as
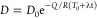
3

Note that eq 3 is based on the fact that the diffusivity of the diffusive species is independent of local concentration of the diffusive species and there is no phase change during the heating.

Considering the periodic characteristic of the multilayer structure, the diffusion problem in the multilayer structure can be simplified to as the diffusion problem in a symmetric trilayer structure (see Figure 1) as follows. For one-dimensional diffusion in the trilayer, the diffusion equation is
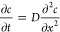
4with *c* being the concentration of the diffusive species and *D* being given in eq 3. The initial condition is
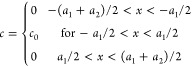
5

The symmetric characteristic of the problem yields the boundary conditions as
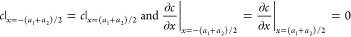
6

Note that the initial condition of (5) can also be used for the outer layers with uniform distribution of the diffusive species, *c*_s0_, by introducing an auxiliary variable of *c̃* (=*c* – *c*_s0_).

Using the method of separation of variables, the concentration of the diffusive species can be expressed as
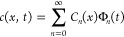
7in which *C_n_*(*x*) and Φ_*n*_(*x*) satisfy the following differential equations
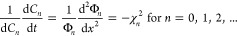
8

The solutions of Φ_*n*_(*x*) and *C_n_*(*x*), which satisfy the boundary conditions of (6), are
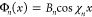
9
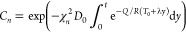
10with *B_n_* being the coefficients to be determined from the initial condition. The eigenvalues of χ_*n*_ are
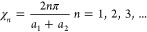
11

Using the initial condition of (5), the coefficients *B_n_* are found to be
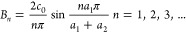
12

Thus, the spatiotemporal evolution of the concentration of the diffusive species is
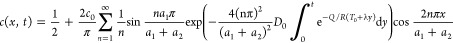
13in which 1/2 is included to satisfy the condition of *c*(*x*, 0) = 0 for *a*_1_/2 < |*x*| < (*a*_1_ + *a*_2_)/2.

According to Mizoguchi and Murata^29^ and Schmidt et al.,^37^ the principles of using X-ray and NR in the characterization of the diffusion coefficient of the diffusive species is that the intensities of the X-ray and NR Bragg peaks are proportional to the square of the coefficient of cos χ*_n_x* in eq 13. Thus, we have
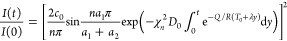
14which gives
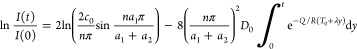
15

It is evident that eq 15 is slightly different from eq 2 with the contribution of a constant term to the peak intensity of X-rays or neutrons.

Generally, it would be relatively easy to curve-fit experimental results with an explicit formulation. The integration in eq 15 increases the complexity in the curve fitting, and a simple polynomial relation between *I*(*t*) and *Q* is desirable. Expanding the exponential term in eq 15 in a Taylor series, eq 15 can be expressed as
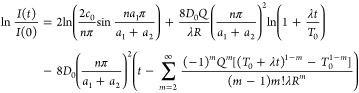
16

To the order of *t*^4^, the temporal variation of ln[*I*(*t*)/*I*(0)] is found to be
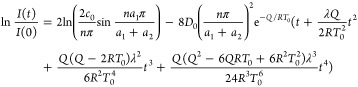
17

A nominal diffusion time τ is introduced and defined as
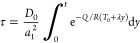
18

**Figure 2:**
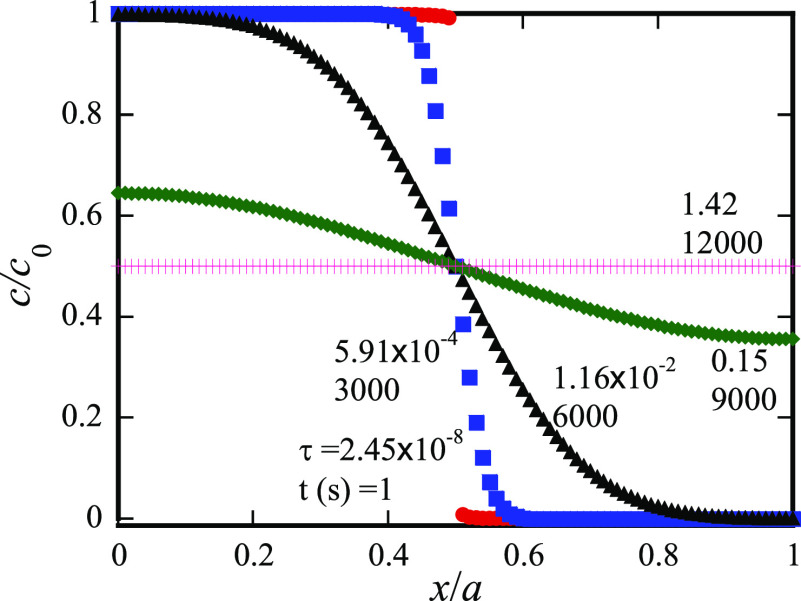
Spatial distribution of the diffusive species (Ge in this case) for *a*_1_ = *a*_2_ = 165 Å at different instants.

Hüger et al.^55^ reported *Q* = 2.14 eV (207.4 kJ/mol) and *D*_0_ = 1.25 × 10^–5^ m^2^/s for the self-diffusion of Ge in a multilayer structure with 2 min in the counting time for a single reflectivity pattern. The multilayer structure consisted of 10 bilayers of ^73^Ge (165 Å)/^nat^Ge (165 Å), which was annealed at a ramping rate of 1 °C/min in a temperature range of 320–500 °C. Using their data and eq 13, the spatial distribution of the diffusive species (Ge in this case) is shown in Figure 2 for *a*_1_ = *a*_2_ = 165 Å at different instants. At the diffusion time of *t* = 1 s, there is limited diffusion occurring at the position of *x* = *a*_1_/2 with the concentration of the diffusive species at *c*_0_ in the most portion of the region [0, *a*_1_/2) and at 0 in the most portion of the region (*a*_1_/2, *a*_2_/2]. Increasing the diffusion time leads to that more diffusive species migrate across the cross-section of *x* = *a*_1_/2, resulting in the decrease of the total amount of the diffusive species in the region [0, *a*_1_/2) and the increase of the total amount of the diffusive species in the region (*a*_1_/2, *a*_2_/2], and finally to the equilibrium state with uniform distribution of the concentration of the diffusive species.

**Figure 3:**
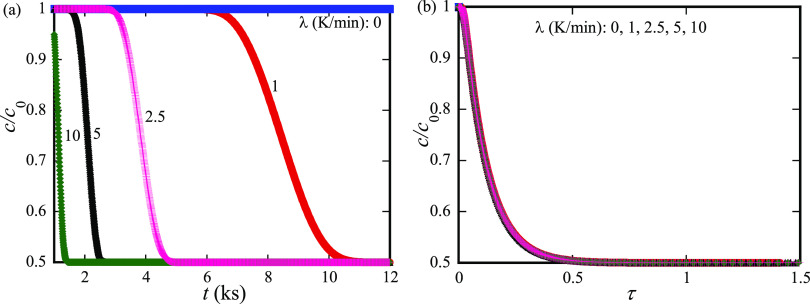
(a) Temporal evolution of the concentration of the diffusive species at the center of the trilayer (*x* = 0) for five different heating rates of 0, 1, 2.5, 5, and 10 K/min over a period of 12 ks (3.33 h) and (b) variation of the concentration of the diffusive species at the center of the trilayer (*x* = 0) with nominal diffusion time for five different heating rates of 0, 1, 2.5, 5, and 10 K/min.

Figure 3a shows temporal evolution of the concentration of the diffusive species at the center of the trilayer (*x* = 0) for five different heating rates of 0, 1, 2.5, 5, and 10 K/min over a period of 12 ks (3.33 h). Without the increase in temperature (λ = 0), there is no observable change in the concentration of the diffusive species over a period of 12 ks. Increasing the heating rate causes the increase in the temperature of the trilayer and accelerates the diffusion of the diffusive species. The time for the onset of the migration of the diffusive species away from the center of the trilayer decreases with increasing the heating rate due to the increase in the diffusivity.

Figure 3b presents the temporal evolution of the concentration of the diffusive species at the center of the trilayer (*x* = 0) with the nominal diffusion time. It is interesting to notice that there is no observable difference of the correlation between the variation of the concentration of the diffusive species at the center of the trilayer and the nominal diffusion time. Such a result suggests that the effect of the constant heating rate on the diffusion of the diffusive species can likely be uniquely described by using the nominal diffusion time given in eq 18.

**Figure 4:**
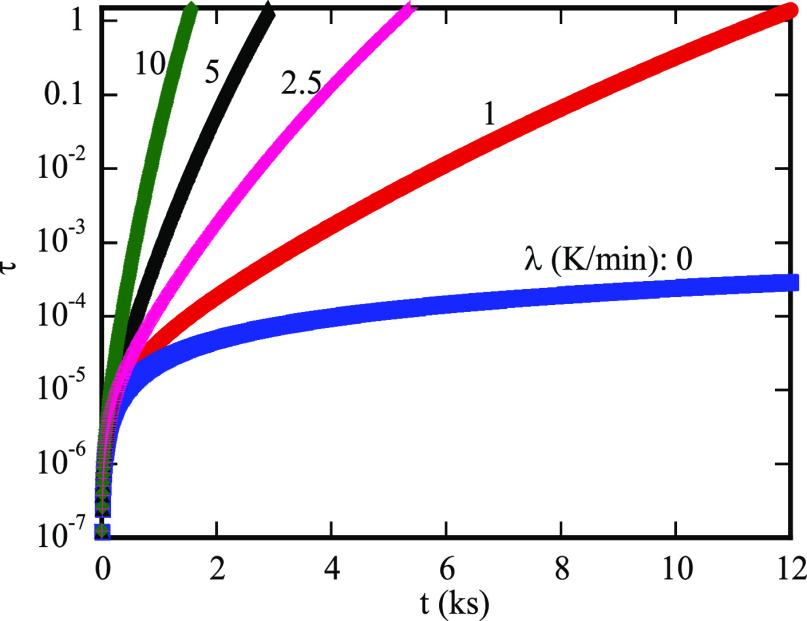
Correlation between the diffusion time and nominal diffusion time.

The variation of the nominal diffusion time with the diffusion time is presented in Figure 4. It is evident that the nominal diffusion time is dependent on the heating rate, as expected. For isothermal heating, there is no significant change in the logarithm of the nominal diffusion time for the diffusion time in the range of 2–12 ks. Note that the nominal diffusion time is proportional to the diffusion time for isothermal heating. The rapid change in the logarithm of the nominal diffusion time occurs for the diffusion time in the range of 0–1 ks, and the range of the diffusion time for the rapid change in the logarithm of the nominal diffusion increases with the increase of the heating rate. Also, the larger the heating rate, the larger the nominal diffusion time at the same diffusion time.

To analyze the variation of the intensities of X-ray and neutron Bragg peaks with the diffusion time, we limit the analysis to the case of *n* = 1. For *n* = 1, eq 16 yields
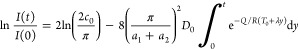
19and eq 17 gives
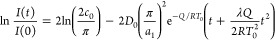
20
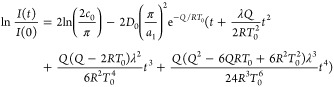
21for the Taylor series expansions of eq 19 to the orders of *t*^2^ and *t*^4^, respectively.

**Figure 5:**
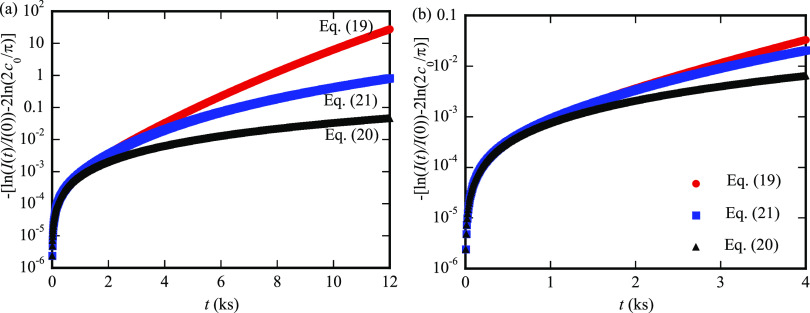
Variation of the intensity of the X-ray or neutron Bragg peak (ln[*I*(*t*)/*I*(0)] – 2ln[2*c*_0_/π]) with the diffusion time in the range of 0–12 ks (a) and in the range of 0–4 ks (b).

Figure 5a shows the variation of the intensity of X-ray or neutron Bragg peak (ln[*I*(*t*)/*I*(0)] – 2ln[2*c*_0_/π]) with the diffusion time in the range of 0–12 ks. For comparison, the numerical results calculated from eqs 20 and 21 are also included in Figure 5a. In general, both eqs 20 and 21 underestimate the change of the intensity of the X-ray or neutron Bragg peak with the diffusion time for large diffusion times, while there exists a range of the diffusion time with the numerical results calculated from eqs 20 and 21 being nearly overlapped with the corresponding ones from eq 19, as shown in the enlarged view in Figure 5b for a small diffusion time. For the diffusion time in the range of 0–1 ks, the results from eq 20 are nearly overlapped with the corresponding ones from eq 19; for the diffusion time in the range of 0–3 ks, the results from eq 21 are nearly overlapped with the corresponding ones from eq 19. These trends imply that eqs 20 and 21 can be likely used to approximately calculate the activation energy and the prefactor for small diffusion times (small range of temperatures).

## Discussion

According to the results shown in Figure 3b, the correlation between the concentration of the diffusive species and the nominal diffusive time defined in eq 18 is independent of the heating rate for the heating rate in the range of 0–10 K/min. Such a result implies that the variation of the intensity of the X-ray or neutron Bragg peak (ln[*I*(*t*)/*I*(0)]) with the nominal diffusion time is likely also independent of the heating rate for the heating rate in the range of 0–10 K/min.

Comparing eq 18 with eq 15, we obtain
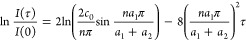
22

The logarithm of the temporal variation of the intensity of the X-ray or neutron Bragg peak (ln[*I*(τ)/*I*(0)]) is a linear function of the nominal diffusion time. Eq 22 reveals that the logarithm of the temporal variation of the intensity of the X-ray or neutron Bragg peak for a multilayer structure under continuous heating with a constant heating rate is a linear function of the nominal diffusion time if the diffusion mechanism remains the same for the temperature range under investigation. Note that such a linear relation is not discussed in the work of Hüger et al.^55^

**Figure 6:**
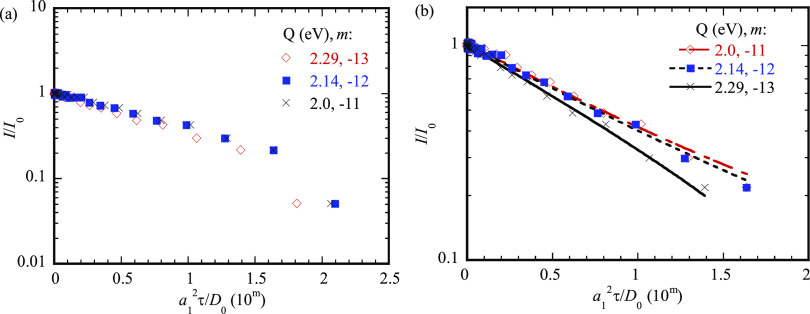
(a) Variation of the integrated and normalized intensity of the Bragg peak reported by Hüger et al.^55^ with the characteristic time for three different activation energies of 2.0, 2.14, and 2.29 eV and (b) linear-regression fitting of the integrated and normalized intensity of the Bragg peak shown in (a) for *I*/*I*_0_ larger than 0.051 with *m* as the power of 10.

Hüger et al.^55^ also reported the temporal evolution of the integrated and normalized intensity of the Bragg peak for the self-diffusion of Ge in the multilayer structure with 5 min in the counting time for a single reflectivity pattern. Figure 6a shows the variation of the integrated and normalized intensity of the Bragg peak of their experimental data with the nominal diffusion time of eq 18 for three different activation energies of 2.0, 2.14, and 2.29 eV. It is evident that the logarithm of the integrated and normalized intensity of the Bragg peak of their experimental data is nearly a linear function of the nominal diffusion time except the last experimental data of *I*/*I*_0_ = 0.051. Figure 6b presents linear-regression fitting of the data shown in Figure 6a for *I*/*I*_0_ larger than 0.051. For the comparison, the fitting curves are also included in the figure. The square of the goodness of fit (*R*) is 0.99 for all the three fitting, which validates the relation of eq 22.

The curve fitting yields 2π^2^*D*_0_/*a*_1_^2^ to be 0.918 × 10^11^, 0.932 × 10^12^, and 1.11 × 10^13^ s^–1^ for the activation energies of 2.0, 2.14, and 2.29 eV, respectively. Thus, the prefactor *D*_0_ is found to be 1.27 × 10^–5^, 1.28 × 10^–4^, and 1.53 × 10^–3^ m^2^/s for the activation energies of 2.0, 2.14, and 2.29 eV, respectively. Both the activation energies and the corresponding prefactors fall in the ranges of the activation energy and the prefactor given by Hüger et al.^55^

**Figure 7:**
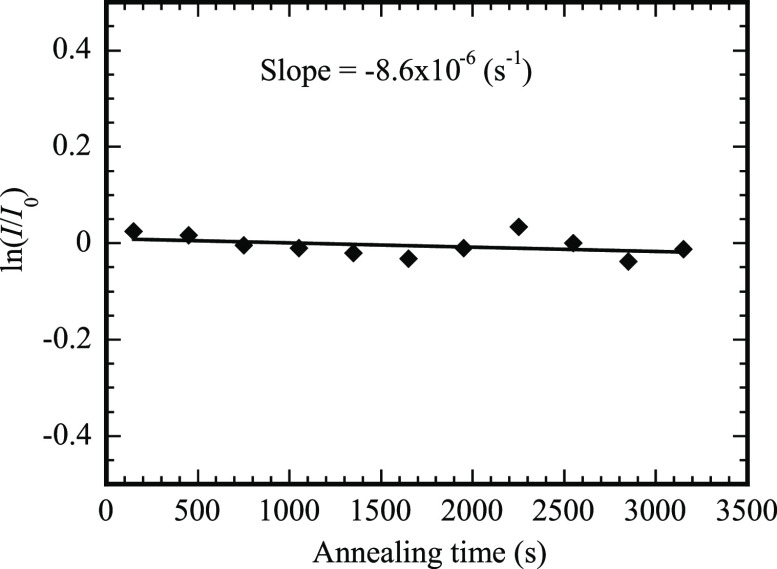
Variation of the integrated and normalized intensity of the Bragg peak of the experimental data with the annealing time less than 3500 s.

As discussed above, eq 20 can be used to approximately calculate the activation energy and the prefactor for small diffusion times (small range of temperatures). Figure 7 shows the variation of the integrated and normalized intensity of the Bragg peak of the experimental data^55^ with the annealing time less than 3500 s. It is evident that the integrated and normalized intensity of the Bragg peak is nearly a linear function of the annealing time. According to eq 20 and the experimental data shown in Figure 7, the term of *t*^2^ in eq 20 is thus negligible. This result suggests that the coefficient of the term of *t*^2^ is much less than the corresponding one for the term of *t* in agreement with the condition for the Taylor series expansion. Such a linear relation between the integrated and normalized intensity of the Bragg peak of the experimental data and the annealing time implies that one can use the linear relation to determine the activation energy from the ramp heating for a small annealing time as follows.

For a small annealing time, the linear relation between the integrated and normalized intensity of the Bragg peak and the annealing time can be expressed as
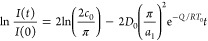
23

For different starting temperatures of the annealing processes of the multilayer structure, the coefficient of the term of *t* is an exponential function of the starting temperature. Defining χ as the coefficient of the term of *t*, we have
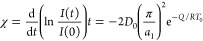
24which gives
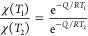
25with *T*_1_ and *T*_2_ being the starting temperatures of two different annealing processes. From the temporal variation of the integrated and normalized intensity of the Bragg peak for small annealing times, we can find the coefficients of the term of *t* for the two different annealing processes and then calculate the activation energy of the diffusion process in the multilayer structure from eq 25. Using linear-regression fitting to fit the experimental data in Figure 7, we obtain a slope of −8.6 × 10^–6^ s^–1^, i.e., χ(593 K) = −8.6 × 10^–6^ s^–1^, which is comparable to −6.5 × 10^–6^ s^–1^ obtained from eq 24 with *D*_0_ = 19.95 × 10^–4^ m^2^/s and *Q* = 2.20 eV. Note that the numerical values of *D*_0_ = 19.95 × 10^–4^ m^2^/s and *Q* = 2.20 eV fall in the ranges of *D*_0_ = 1.55_–1.3_^+18.4^ × 10^–4^ m^2^/s and *Q* = 2.29_–0.15_^+0.17^ eV given by Hüger et al.^55^

It is worth mentioning that the experimental data of *I*/*I*_0_ presented in Figures 6 and 7 were obtained by subtracting the background of all the measured Bragg peaks, i.e., the experimental data of *I*/*I*_0_ presented in Figures 6 and 7 do not contain any information of the first term on the right-hand side of eqs 22 and 23. According to eq 24, the information of the first term on the right-hand side of eqs 22 and 23 has no effects on the calculation of the activation energy of the diffusion process in the multilayer structure. Thus, the slope obtained from Figure 7 can be used in the calculation of the activation energy of the diffusion process from eq 24.

It should be pointed out that the above analysis is based on the fact that the multilayer experiences uniform change in temperature during the diffusion annealing under a constant heating rate. There are actually two processes involved in the diffusion annealing of the multilayer structure: one is the heat conduction and the other is the mass transport. For the heat conduction, the characteristic time, *t*_h_, is calculated as
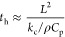
26with *L* being the thickness of the multilayer structure and *k*_c_, ρ, and *C*_p_ the effective thermal conductivity, effective density, and specific heat at constant pressure of the multilayer, respectively. For the diffusion, the characteristic time, *t*_d_, is calculated as
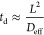
27with *D*_eff_ being the effective diffusion coefficient of the diffusive species in the multilayer structure.

To have a uniform distribution of temperature under the diffusion annealing for the in situ measurement of the activation energy for the diffusion of the diffusive species, it requires
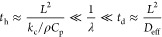
28in which _f_*t*_h_ and *t*_d_ are the lower and upper bounds of the characteristic time for diffusion annealing in the in-situ measurement. Eq 28 provides the condition of determining the heating rate applicable to the in situ measurement of the activation energy for the diffusion of the diffusive species in a multilayer structure.

For the multilayer structure used by Hüger et al.,^55^ there are 10 bilayers of ^73^Ge (165 Å)/^nat^Ge (165 Å) and *L* = 0.33 μm. For germanium, *k*_c_ = 58 W/m·K, *C*_p_ = 320 J/kg·K, ρ = 5323 kg/m^3^ and *D* = 1.20 × 10^–19^ m^2^/s as calculated from *Q* = 2.14 eV (207.4 kJ/mol), *D*_0_ = 1.25 × 10^–5^ m^2^/s, and *T*_f_ = 793 K. We have *t*_h_ = 3.2 × 10^–9^ s and *t*_d_ = 908,010 s. For the heating rate of 1–10 K/min, the corresponding heating time per degree is 60 to 6 s, which satisfies eq 28. Thus, the use of uniform temperature in the multilayer under the heating condition used in the analysis is reasonable.

## Summary

Mass transport plays an important role in the formation and growth of new phases and new compounds, which determine the structural integrity of multilayer structures used in a variety of optoelectronic devices and solid-state energy storage.^2−4^ One of the challenges in understanding mass transport on micro- and nanoscales is the determination of the activation energy responsible for the rate process controlling the mass transport. The works presented by Mizoguchi and Murata^29^ and extended by Hüger et al.^55^ point to the feasibility of determining the activation energy for atomic diffusion in multilayer structures by using a constant heating rate during the annealing process if there are no changes in the activation energy for diffusion.

We have analytically solved the diffusion problem in a multilayer structure with periodic conditions under a constant heating rate. Correlating the coefficients of eigenfunctions with the intensity of the X-ray or neutron Bragg peak establishes the analytical expression for the determination of the activation energy from the annealing under a constant heating rate. Introducing a nominal diffusion time of eq 18, the logarithm of the temporal variation of the intensity of the X-ray peak or Bragg peak for a multilayer structure under continuous heating with a constant heating rate is found to be a linear function of the nominal diffusion time if the diffusion mechanism remains the same for the temperature range under investigation. The experimental results given by Hüger et al.^55^ support the linear relationship used to determine the activation energy for the diffusion in multilayer structures. From this relationship, a simple method is proposed for the determination of the activation energy of the diffusion in multilayer structures under a small annealing time.

It should be pointed out that the method presented by Mizoguchi and Murata^29^ and extended by Hüger et al.^55^ is based on the uniform distribution of temperature in the multilayer structure. This requires that the characteristic time for heat conduction is much less than the characteristic time for the ramp heating as well as the characteristic time for diffusion, as shown in eq 28.
